# The Influence of Exercise-Induced Fatigue on Inter-Limb Asymmetries: a Systematic Review

**DOI:** 10.1186/s40798-020-00270-x

**Published:** 2020-08-26

**Authors:** Jessica Heil, Florian Loffing, Dirk Büsch

**Affiliations:** Institute of Sport Science, Carl von Ossietzky University Oldenburg, Ammerländer Heerstraße 114-118, 26129 Oldenburg, Germany

**Keywords:** Physical load, Injuries, Biomechanics, Side-to-side differences

## Abstract

**Background:**

Non-contact injuries such as anterior cruciate ligament ruptures often occur during physical load toward the end of a match. This is ascribed to emerging processes due to exercise-induced fatigue. Moreover, non-contact injuries often occur during dynamic actions such as landing or cutting movements. Inter-limb asymmetries are suggested as one possible cause for those injuries based on findings indicating that asymmetries between limbs are associated with a higher injury risk. Hence, assessing inter-limb asymmetry during physical load in the condition of exercise-induced fatigue is warranted to identify potentially relevant precursors for non-contact injuries.

**Objective:**

The objective of this study was to overview the current state of evidence concerning the influence of exercise-induced fatigue on inter-limb asymmetries through a systematic review.

**Methods:**

A systematic literature search was conducted using the databases Web of Science, Scopus, PubMed, SURF, and SPONET to identify studies that assessed inter-limb asymmetries of healthy people, calculated with an asymmetry equation, before and after, or during a loading protocol.

**Results:**

Thirteen studies were included in the systematic review. The loading protocols involved running, race walking, jumping, squatting, soccer, rowing, and combinations of different exercises. Moreover, different tasks/procedures were used to assess inter-limb asymmetries, e.g., squats, single-leg countermovement jumps, gait analysis, or isokinetic strength testing. The results seem to depend on the implemented loading protocol, the tasks/procedures, and the measured parameters.

**Conclusions:**

Future research needs more systematization and consistency, assessing the effect of exercise-induced fatigue on inter-limb asymmetries. Moreover, the emergence of inter-limb asymmetries should be regarded in the context of sport-specific movements/tasks. Testing before, after, and during a physical loading protocol is advisable to consider the influence of exercise-induced fatigue on sport-specific tasks and to identify the possible mechanisms underlying load-dependent inter-limb asymmetries with regard to risk of non-contact injury.

## Key Points

- Inter-limb asymmetries and exercise-induced fatigue are suggested risk factors for non-contact related injuries.
- The amount of inter-limb asymmetries during physical load is influenced by different factors, e.g., the used tasks/procedures, the implemented loading protocols, or the measured parameters.
- Research on the influence of exercise-induced fatigue on inter-limb asymmetries needs more systematization to provide better insight into the suggested causal relationship and its underlying mechanisms.

## Background

Athletes must deal with different external physical loads during training, matches, or competition. Physical loads are the entirety of the ascertainable influences in the training system that affect the athlete. These impacts lead to different internal loads depending on the individual characteristics of an athlete [1]. In sports, physical loads are necessary to improve or maintain an athlete’s performance [1, 2]. At the same time, they also expose athletes to a risk of sustaining an injury by putting them into situations with high impacting forces such as tackling, landing, or cutting. Many injuries, especially non-contact injuries such as anterior cruciate ligament (ACL) ruptures, occur during physical load toward the end of a match [3–5]. Often a single or repetitive physical load causes such injuries [6]. Exercise-induced fatigue, meaning fatigue due to physical load (e.g., training or competition), is one possible consequence potentially enhancing the likelihood for non-contact injuries [7–9]. Specifically, exercise-induced fatigue might alter physiological processes, thereby reducing, for example, the level of voluntary muscle activation or altering muscle activation patterns [8].

Furthermore, non-contact injuries often occur during dynamic actions such as landing or cutting [10, 11]. One reason suggested underlying this phenomenon are differences between an individual athlete’s limbs, i.e., inter-limb asymmetries [12]. Inter-limb asymmetries relate to the phenomenon of reduced function, physical capacity, strength, etc. of one limb in relation to the other. In sports, inter-limb asymmetries might be functionally induced as a consequence of the sporting activity [13–15], especially in sports that are mainly characterized by asymmetric (or unilateral) execution of movements with the preferred limb such as kicking in soccer or throwing in handball [16]. However, also in symmetric sports that are characterized by cyclic or alternating movement patterns (e.g., running, cycling, or swimming), inter-limb asymmetries occur [13, 15, 17]. Those asymmetries might originate from the preference for one side of the body over the other. According to Parrington et al. [17], the predominant use of the limb “on one side of the body can cause uneven flexibility, range of movement, strength development, and neural development occurring on the favored side” (p. 283), ultimately leading to inter-limb asymmetries.

Inter-limb asymmetries are associated with a higher injury risk because they might lead to unequal force absorption or a loss of frontal plane stability, which are essential to bear the impacting forces [18]. Most commonly, differences of 10 to 15% in parameters such as ground reaction force (GRF), impulses or jump height, between limbs are said to be critical [19], and often these values are also used as criteria for a return to play decision following the recovery from injury [20, 21]. However, these limits are criticized for being arbitrary and needing better empirical justification [19]. Nevertheless, it must be mentioned that the concept of thresholds in the context of inter-limb asymmetries must be doubted [22].

Assessing the impact of exercise-induced fatigue on inter-limb asymmetries appears of particular importance with regard to understanding the mechanisms possibly underlying non-contact injuries. Exercise-induced fatigue may evoke otherwise non-apparent or worsen pre-existing inter-limb asymmetries [7, 17, 23] potentially driven by worse movement patterns due to detrimental changes of neuromuscular control, altered proprioception, postural control, or movement coordination due to fatigue [23]. These processes might influence the execution of certain movements or parameters of a movement leading to or worsening asymmetries [17]. A systematic overview and discussion of evidence on whether and how inter-limb asymmetries change under the influence of exercise-induced fatigue are missing so far. Here, we aimed to systematically review the current findings concerning the influence of exercise-induced fatigue on inter-limb asymmetries.

## Methods

### Literature Search

A systematic literature search was conducted in January 2020 according to the Preferred Reporting Items for Systematic Reviews guidelines (PRISMA) [24] to review the current state of evidence concerning the influence of exercise-induced fatigue on inter-limb asymmetries. The search was performed in five different databases: Web of Science (all databases), Scopus, PubMed, SURF (in German and English), and SPONET (in German and in English). The search strategy included a combination of terms concerning (1) load/fatigue, (2) asymmetries, and (3) limbs (Table 1).

**Table 1:** Search strategy (English version)

Operator		Terms
	#1	fatig* OR exhaust* OR weari* OR tired* OR exert* OR stress OR load OR strain OR effort
AND	#2	asymmetr* OR imbalance* OR dissymmetr* OR “side-to-side difference” OR “side difference” OR “lateral difference”
AND	#3	limb* OR arm* OR leg* OR thigh OR knee OR hip OR ankle OR calf OR shoulder

*truncation character

The results of the different searches were combined, and the duplicates were removed. The titles and abstracts of the remaining articles were checked, and irrelevant articles were excluded. Afterward, the full texts of the suitable articles were analyzed for eligibility. Additionally, a manual check of the list of references of the included studies was performed.

To be eligible for the systematic review, the studies had to match each of the following criteria: (1) investigated the influence of exercise-induced fatigue on biomechanical (kinetic or kinematic) parameters in a sports-related context, (2) compared the measured values between the limbs by calculating asymmetry or asymmetry could be calculated post-hoc based on the values provided in an article, (3) recorded the values at a minimum of two points of time before and after or during the progression of a generalized loading protocol that affects the cardiovascular and motor systems in whole, (4) included healthy subjects, and (5) full text available in English or German. Reviews, abstracts, project descriptions, conference papers, interviews, theoretical papers, or dissertations were excluded. The year of publication was not restricted.

### Data extraction and analysis

The central features of the studies were extracted, including subject characteristics, study design, loading protocol details, tasks/procedures to investigate asymmetries, asymmetry equation, outcome measurements, and results. For those studies that did not report effect sizes or asymmetry indices, the corresponding values were calculated post-hoc. For missing asymmetry indices, the percentage difference suggested by Bishop et al. [25] was calculated according to the formula: 100/ (max value) × (min value) × (− 1) + 100. For missing effect sizes, Cohen’s *d* [26] was calculated as suggested by Cumming [27] for dependent samples using: *d* = *M*_diff_/SD_pre_, where *M*_diff_ is the difference of the mean values and SD_pre_ the standard deviation of the pretest scores. The effect sizes were categorized as either small (0.2 ≤ *d* < 0.5), medium (0.5 ≤ *d* < 0.8), or large (*d* ≥ 0.8) according to Cohen [26]. No meta-analysis could be performed due to the heterogeneity of the included studies concerning the loading protocols, the tasks/procedures, and due to the different calculations of asymmetry and the missing of necessary values to compute post-hoc effect sizes.

### Study Quality

The methodological quality of the included studies was evaluated with a scale extracted from the systematic review by Bishop et al. [19]. These authors adjusted the scale from a systematic review of Black et al. [28]. The scale was originally developed by Brughelli et al. [29] for studies in the field of sports and exercise training. The scale consisted of nine different criteria that were rated with 0 = no, 1 = maybe, and 2= yes (Table 2).

**Table 2:** Study quality scoring system (adapted from [19])

Criteria	Item	Score
1	Inclusion criteria stated	0–2
2	Subjects assigned appropriately	0–2
3	Procedures described	0–2
4	Dependent variables defined	0–2
5	Assessments practical	0–2
6	Training duration practical (acute vs. long term)	0–2
7	Statistics appropriate	0–2
8	Results detailed (mean, standard deviation, percent change, effect size)	0–2
9	Conclusions insightful (clear, practical application, future directions)	0–2
Total		0–18

## Results

### Database Search

**Fig. 1:**
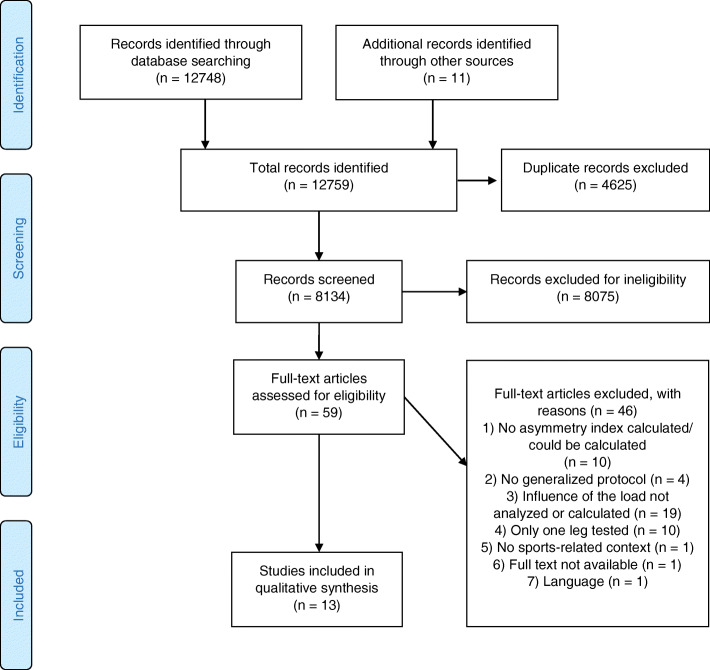
Flowchart of the searching process (from [24])

In total, the searches revealed 12,748 articles (Web of Science = 6106, Scopus = 4580, PubMed = 1498, SURF = 357, SpoNET = 207), thereof 4625 duplicates were removed. Additional searches revealed eleven articles. The remaining 8134 were checked by title and abstract for their eligibility according to the above-mentioned criteria. This resulted in 59 articles for further full text inspection, which finally led to 13 articles being included in the review. Reasons for exclusion based on analysis were (1) no asymmetry equation was used or could be used to calculate the values afterwards (*n* = 10), (2) no generalized loading protocol (*n* = 4), (3) influence of exercise-induced fatigue on asymmetries was not analyzed or compared at different points of time (*n* = 19), (4) only one leg tested (*n* = 10), (5) no sports-related context (*n* = 1), (6) full text not available (*n* = 1), and (7) language (*n* = 1) (Fig. 1).

### Study Characteristics

#### Study Design

The studies used different designs. A differentiation can be made between studies regarding inter-limb asymmetries during a loading protocol [20, 21, 30–34] (Table 3) and studies regarding inter-limb asymmetries with a supplementary task in a pre-post design [7, 16, 35–38] (Table 4). In six studies, the subjects were tested for asymmetries at different points of time during the loading protocol [20, 21, 31–34]. One study interspersed the loading protocol with several single-leg countermovement jumps (SLCMJ) to test asymmetries [30], and six studies used other tasks/procedures, such as jumps, isokinetic strength testing or a gait analysis, to test the subjects before and after the loading protocol [7, 16, 35–38]. Furthermore, all studies investigated the acute influence of exercise-induced fatigue on inter-limb asymmetries. Only Bromley et al. [16] additionally considered specific points of time after the physical load to evaluate not only the effect of acute fatigue but also asymmetries during the recovery period. Moreover, Bishop et al. [38] tested asymmetries with several jump tests before and after five consecutive soccer matches to investigate long term changes.

**Table 3:** Study characteristics—asymmetry examined during a loading protocol/task

Study	Subjects (Sex, age, sport)	Physical load/fatigue loading protocol	Measure of fatigue	Task/procedure	Outcome measures	Asymmetry equation
Bishop et al. [30]	*n* = 18 18 M (28.9 ± 5. 1) Recreationally active	4 rounds of 6 × 40 m sprints on running track (interspersed with a SLCMJ)	Fatigue index = (100 × (total sprint time/ideal sprint time)) - 100)	SLCMJ	Jump height	Percentage difference: 100 / (maximum value) × (minimum value) × − 1 + 100
Girard et al. [20]	*n* = 13 13 M (31.2 ± 4.8) Recreational team- and/or racket-sport	RSA test	Sprint decrement score	Running on a treadmill	Running kinetics, running kinematics and spring-mass characteristics	Bilateral leg asymmetry (BLA%): [(high value − low value)/ low value] × 100
Hanley and Tucker [31]	*n* = 14 14 M (31 ± 7) Competitive runners (best time 10 km 31:00–35:20)	10,000 m run (103% of season's best)	RPE scale by Borg	Running on a treadmill	vGRF, spatio-temporal data (step length, step frequency, contact time, flight time, impact force, maximum force, impulse), gait variability	Symmetry angle: [(45° - arctan (X_left_/X_right_)/90°)] × 100% X = the mean value for a variable on each leg
Hodges et al. [32]	*n* = 17 9 M / 8 F (22.3 ± 2.5) Recreationally trained	5 sets with 8 repetitions of free-weight barbell back squats, 3 min rest between each set, 90% of 8 repetitions maximum	Not measured	Free-weight barbell back squats	vGRF	GRF asymmetry: left vGRF% - right vGRF%
Jordan et al. [21]	*n* = 22 12 M, 10 F 11 ACLR (age N/A) 6 M (26.5 ± 5.8) / 5 F (23.6 ± 1.8) 11 CG (age N/A) 6 M (23.3 ± 3.3 / 5 F (21.8 ± 3.2) Elite skiers	Jump test loading protocol (20 squat jumps in 80 s)	Operational definition of fatigue: exercise-induced decrease in lower limb muscle power	Jump test (Squat jumps)	Vertical ground reaction force (Fz), EMG data, vertical jump HBCM	Asymmetry Index control: [(left limb impulse – right limb impulse) / (maximum of left and right impulse)] × 100 Asymmetry Index ACLR: [(contralateral limb impulse – affected limb impulse) / (maximum of contralateral and affected limb impulse)] × 100
Mattes and Wolf [33]	*n* = 32 16 M, 16 F (age N/A) High-performance junior rowers (German National Team U19)	2000-meter race test	Not measured	On-water rowing test	Leg stretcher force	Symmetry Index: [(x_1_ - x_2_) / 0.5 (x_1_ + x_2_)] × 100% x_1_ = outside leg x_2_ = inside leg
Tucker and Hanley [34]	*n* = 10 5 M / 5 F (age N/A for the subgroup) Elite race walkers	10 km race-walk on treadmill − 103% of their most recent 20 km speed	Not measured	Race walking on a treadmill	vGRF data (impact peak force, loading peak force, mid-stance force, push off peak force, impulse)	Symmetry angle: [(45° - arctan (X_left_/X_right_)/90°)] × 100% X = the mean value for a variable on each leg

*ACLR* anterior cruciate ligament rupture, *BLA* bilateral leg asymmetry, *CG* control group, *EMG* electromyography *F* female, *GRF* ground reaction forces, *HBCM* height of body center of mass, *M* male, *N/A* not available, *RPE* rate of perceive exertion*, RSA* repeated sprint ability, *SLCMJ* single-leg countermovement jump, *vGRF* vertical ground reaction force

**Table 4:** Study characteristics—asymmetries examined pre and post a loading protocol/task

Study	Subjects (Sex, age, sport)	Physical load/fatigue loading protocol	Measure of fatigue	Task/procedure	Outcome measures	Asymmetry equation
Bell et al. [7]	*n* = 40 20 M (20.9 ± 1.2)/ 20 F (21.2 ± 1.4) Recreationally active	Exercise loading protocol based on literature: warm-up–running course––30 s wall sit–10 fast-paced two-legged vertical jumps––30 s prone plank	Loading protocol was repeated until a RPE of 17 on the scale by Borg was reached	Jump landings	Peak vGRF, loading rate, LESS score	% asymmetry: ([dominant limb – non-dominant limb]/ 1/2 [dominant limb + non-dominant limb]) × 100%
Bishop et al. [38]	*n* = 18 18 M (16.89 ± 0.32) Elite academy soccer players	Five Soccer matches (played for a minimum of 60 min in each match)	Not measured	SLCMJ, SLDJ	SLCMJ: jump height, peak force, concentric impulse SLDJ: jump height, ground contact time, RSI Global positioning system data	Percentage difference: 100/ (maximum value) × (minimum value) × -1 + 100
Bromley et al. [16]	*n* = 14 14 M (17.6 ± 0.5) Elite soccer players	Single 90-min soccer match	Not measured	SLCMJ	Jump height, peak force, eccentric impulse, concentric impulse, peak landing force, peak landing impulse	Percentage difference: 100/ (maximum value) × (minimum value) × − 1 + 100
Delextrat et al. [37]	*n* = 14 14 F (26.1 ± 4.6) Amateur soccer players	Modified Loughborough Intermittent Shuttle Test	RPE	Isokinetic strength assessment	Peak torque of the quadriceps, peak torque of the hamstrings, H_ecc_:Q_con_	Calculated afterwards by the authors with percentage difference: 100/ (maximum value) × (minimum value) × − 1 + 100
Radzak et al. [35]	*n* = 20 14 M / 6 F (20.8 ± 2.48) No sporting background mentioned	Speed blinded exhaustive loading protocol	RPE Until volitional exhaustion	Gait analysis	Kinematic data (joint angles - ankle, knee, hip), kinetic data (external joint moment - ankle, knee, hip), GRF, stiffness, spatio-temporal parameters	Symmetry angle: [(45° - arctan (X_left_/X_right_)/90°)] × 100% X = the mean value for a variable on each leg
Webster et al. [36]	*n* = 20 20 M 10 ACLR (23 ± 3) 10 CG (23 ± 2) Engaged in sports activities weekly	Generalized loading protocol: squats 10 ×, two vertical jumps, 10 drop landings (5 right/ 5 left) (repeated several times)	Fatigue was operationally defined: jump height reduced by 20% OR when the subject could no longer complete the fatigue loading protocol	Squats	Kinetic data (external joint moments), ground reaction force (peak vGRF) − weight-bearing symmetry, kinematic data (joint angles)	Symmetry Index = [v_affected_ – v_unaffected_ / ½ (v_affected_ + v_unaffacted_)] × 100 v_affected_ = value of the the former injured leg v_unaffected_ = value of the “healthy” leg

*ACLR* anterior cruciate ligament rupture, *CG* control group, *F* female, *GRF* ground reaction force, *H*_ecc_:*Q*_con_ functional ratio of the peak eccentric torque of the hamstrings to the peak concentric torque of the quadriceps, *LESS* landing error scoring system, *M* male, *N*/*A* not available, *RPE* rate of perceived exertion, *RSI* reactive strength index, *SLCMJ* single-leg countermovement jump, *SLDJ* single-leg drop jumps, *vGRF* vertical ground reaction force

#### Loading Protocols

All thirteen studies used different types of physical load: five studies used a running protocol (three on a treadmill [20, 31, 35], one on a running track [30] and one used a soccer-specific running protocol modified with a kicking task [37]), one study used a race walking protocol on a treadmill [34], one study conducted an on-water field test in rowing [33], two studies tested asymmetries before and after soccer matches [16, 38], two studies used the repetition of exercise as loading protocol (squatting [32], jumping [21]), and two studies used a combination of different exercises [7, 36].

The loading protocols had different durations, and different methods were used to determine the end of the protocol: one study [7] used the ratings of perceived exertion (RPE) scale by BORG [39], two studies ended their loading protocol when the exercise or a certain output could no longer be maintained [21, 36], in two cases the loading protocol was executed until volitional exhaustion [32, 35], and in seven studies the subjects had to complete the prescribed protocol [16, 20, 30, 31, 33, 34, 37, 38]. Moreover, exercise-induced fatigue was inferred based on four methods: four studies [7, 31, 35, 37] used the RPE scale [39], another calculated a fatigue index [30], Girard et al. [20] calculated a sprint decrement score, and others operationalized fatigue as the decrease of lower limb muscle power [21] or the point when an exercise could no longer be maintained [36]. Furthermore, five studies did not measure exercise-induced fatigue and assumed that the physical load was fatiguing [16, 32–34, 38].

#### Tasks/Procedures

Six studies used different tasks/procedures to test the subjects before and after a loading protocol. Bishop et al. [30] interspersed the protocol with a separate task. The tasks/procedures that were used were jump landings [7], SLCMJ [16, 30, 38], single-leg drop jumps (SLDJ) [38], squats [36], gait analysis [35], and isokinetic strength testing [37]. Six studies used no separate task/procedure and measured certain parameters during the loading protocol. Thereby, asymmetries were measured during a repeated sprint ability (RSA) protocol on a treadmill [20], a 10,000-m run or race walk (103% of season's best) on a treadmill [31, 34], a 2000-m on-water rowing test [33], five sets of squats (8 repetitions at 90% of 8 repetitions maximum) [32], or a protocol of 20 squat jumps in 80 s [21].

#### Subject Characteristics

In total, 252 people were examined (173 males and 79 females). Six studies included only male subjects (*n* = 97) [16, 20, 30, 31, 36, 38], six both sexes (76 males, 65 females) [7, 21, 32–35], and one only considered females (*n* = 14) [37]. The number of subjects in a study ranged from ten (five males, five females) [20] to 40 (20 males, 20 females) [7]. Most of the subjects had a sporting background: in five studies, they were recreationally or physically active (*n* = 108) [7, 20, 30, 32, 36], one tested active runners (*n* = 14) [31], one included elite race walkers (*n* = 10) [34]; three studies focused on soccer players (*n* = 46) [16, 37, 38], one on university soccer players (*n* = 14) [37] and two on elite soccer players (*n* = 32) [16, 38]; one study included elite skiers (*n* = 22) [21]; and one was on high-performance junior rowers (*n* = 32) [33]. Radzak et al. [35] tested healthy and injury free athletes (*n* = 20), but did not mention their sporting background. Two of the thirteen studies compared healthy subjects with a former ACL rupture (*n* = 21) with healthy controls without a former ACL rupture [21, 36]. None of the other studies used a control group.

### Study Quality

The study quality was assessed regarding the procedures, statistics, and results on inter-limb asymmetries. All studies reached 14 to 17 points on the quality scoring system (Table 5). Most of the studies clearly described their procedures, defined their variables, and used practical assessments and appropriate statistics. However, the analyses and the report of the results concerning the effect of fatigue on asymmetries were sometimes inadequate. Some studies did not achieve the highest score on a criterion due to the following reasons: no consideration of percentage asymmetry values in their statistical analyses [20, 37], they omitted some mean values and standard deviations on asymmetries [16, 20, 21, 30, 32, 34, 38], effect sizes regarding the effect of fatigue on asymmetries were not calculated or reported [7, 20, 21, 31, 32, 34, 35, 37, 38], and a percent change was not reported in any of the studies.

**Table 5:** Assessment of study quality

Criteria	Item	Bell et al. [7]	Bishop et al. [30]	Bishop et al. [38]	Bromley et al. [16]	Delextrat et al. [37]	Girard et al. [20]	Hanley and Tucker [31]	Hodges et al. [32]	Jordan et al. [21]	Mattes and Wolf [33]	Radzak et al. [35]	Tucker and Hanley [34]	Webster et al. [36]
1	Inclusion criteria stated	2	2	2	2	2	2	1	2	2	1	2	1	2
2	Subjects assigned appropriately	2	2	2	2	2	2	2	2	2	2	2	2	2
3	Procedures described	2	2	2	2	2	2	2	2	2	2	2	2	2
4	Dependent variables defined	2	2	2	2	1	2	2	2	2	2	2	2	2
5	Assessments practical	2	2	2	2	2	2	2	2	2	2	2	2	2
6	Training duration practical (acute vs. long term)	2	2	2	2	2	2	2	2	2	2	2	2	2
7	Statistics appropriate	2	2	2	2	2^a^	1	2	2	2	2	2	2	2
8	Results detailed (mean, standard deviation, percent change, effect size)	1	1	0	1	2^a^	0	1	0	0	2	1	0	1
9	Conclusions insightful (clear, practical application, future directions)	2	2	2	2	2	2	1	2	1	2	2	1	1
Total score		17	17	16	17	17	15	15	16	15	17	17	14	16

^a^Values calculated post-hoc by the authors

### Outcome Analysis

In the studies, different outcome parameters were measured. In all studies, parameters of the lower limbs were regarded, and no study considered the upper limbs. In total, seven different equations were used to calculate asymmetries: two studies used the symmetry angle according to Zifchock and Davis [40], three used the percentage difference suggested by Bishop et al. [25], and the other studies used different equations (see Tables 3 and 4 for details). For the study of Delextrat et al. [37], a percentage difference [25] was calculated and analyzed post-hoc.

Measured parameters for which an asymmetry equation was applied were, among others, GRF [7, 16, 20, 31, 32, 34–36], jump height [16, 30, 38], leg stiffness [20, 35], or leg stretcher force [33]. Tables 3 and 4 show a detailed list of the measured parameters and asymmetry indices. The analysis of the study results (Tables 6 and 7) showed statistically significant differences between the asymmetry values in one study [30]; in five studies, both significant and non-significant results were found [16, 32, 33, 35, 36], varying between the measured parameters. In three studies, the changes were only significant when the group was divided into subgroups [32, 36] or by sex [33], and seven studies found no significant changes due to the implemented loading protocol [7, 20, 21, 31, 34, 37, 38].

**Table 6:** Study results—asymmetries examined during a loading protocol/task

Study	Parameter		t1		t2		t3		t4		t5		*p*	ES (*d*)	
			Mean	SD	Mean	SD	Mean	SD	Mean	SD	Mean	SD			
Bishop et al. [30]	Jump height		7.62	N/A	9.82	N/A	9.95	N/A	13.25	N/A	14.67	N/A	< 0.05	0.83	t1 vs. t4
													< 0.05	1.16	t1 vs. t5
Hanley and Tucker [31]	Step length		0.42	0.39	0.51	0.46	0.53	0.42	0.53	0.45	0.54	0.39	> 0.05 for all variables	0.31*	t1 (1500 m) vs. t5 (9000 m)
	Step frequency		0.58	0.52	0.56	0.36	0.55	0.49	0.56	0.53	0.59	0.46		0.02*	
	Contact time		0.42	0.35	0.33	0.20	0.36	0.22	0.45	0.34	0.46	0.34		0.11*	
	Flight time		1.16	0.92	1.14	0.75	1.25	1.03	1.40	1.20	1.29	1.10		0.14*	
	Impact force		1.97	1.47	2.87	1.52	2.74	2.10	2.65	2.23	2.77	2.37		0.54*	
	Maximum force		1.00	0.81	0.95	0.70	1.01	0.81	1.15	0.87	1.12	0.75		0.15*	
	Impulse		0.86	0.58	0.82	0.59	0.92	0.61	0.73	0.46	0.81	0.65		0.09*	
Matthes and Wolf [33]	Leg stretcher force	Male	8.2	5.9							9.1	7.0	> 0.05	0.15*	60–90 s vs. 360–390 s (Female) / 60–90 s vs. 300–330 s (Male)
		Female	24.9	19.8							28.3	19.5	0.023	0.17*	
Girard et al. [20]	Performance and running kinetics, running kinematics, spring-mass characteristics	Only averaged asymmetry values over all 5 sets of sprints											> 0.05 for all variables	N/A	Could not be calculated
Hodges et al. [32]	absolute average vGRF asymmetry	Not stated											0.6	N/A	Could not be calculated
													0.229		
Jordan et al. [21]	Functional AI values	Not stated											0.76	N/A	Could not be calculated
Tucker and Hanley [34]	Step length, contact time, step frequency, vGRF data	Not stated											> 0.05 for all variables	N/A	Could not be calculated

*AI* asymmetry index, *ES* effect size, *N/A* not available, *t* point of time, *SD* standard deviation, *vGRF* vertical ground reaction force

*value was calculated afterwards by the authors

**Table 7:** Study results—asymmetries examined pre and post a loading protocol/task

Study	Parameter		Pre		Post		*p*	ES (d)	
			Mean	SD	Mean	SD			
Bell et al. [7]	Peak vGRF	Male	16.68	14.53	15.92	14.02	0.94	0.05*	Pre vs. post
		Female	14.55	11.67	14.91	17.81		0.03*	
	Loading rate	Male	25.80	19.64	25.49	18.02	0.43	0.02*	
		Female	19.71	17.28	26.11	25.66		0.37*	
Bishop et al. [38]	SLCMJ	Jump height	Not stated				> 0.05 for all variables	N/A	
		Peak Force							
		CON Impulse							
	SLDJ	Jump height							
		RSI							
Bromley et al. [16]	ECC impulse		14.24	N/A	32.00	N/A	< 0.05	3.15	
	CON impulse		7.73	N/A	10.50	N/A	> 0.05	0.31	
	peak force		14.71	N/A	31.85	N/A	< 0.05	2.8	
	jump height		4.65	N/A	17.22	N/A	> 0.05	1.18	
	peak landing impulse		5.89	N/A	8.51	N/A	> 0.05	0.62	
	peak landing force		7.22	N/A	9.13	N/A	> 0.05	0.32	
Delextrat et al. [37]	Peak torque quadriceps		11.0*	7.2*	13.9*	14.2*	0.51*	0.40*	
	Peak torque hamstrings		13.2*	9.5*	11.8*	7.8*	0.68*	0.15*	
	*H*_ecc_:*Q*_con_		10.4*	8.5*	13.7*	9.6*	0.27*	0.39*	
Radzak et al. [35]	*K*_vert_		4.66	3.28	3.07	2.03	0.034	0.49*	Pre vs. post
	Maximum knee varus velocity		16.97	9.20	13.85	10.24	0.052	0.34*	
	Knee internal rotation excursion		15.37	6.50	29.40	11.81	0.001	2.16*	
	Loading rate		2.96	2.25	1.91	2.25	0.035	0.47*	
	Maximum absolute free moment		10.22	7.27	7.33	5.83	0.057	0.40*	
	Maximum adduction free moment		11.05	8.39	8.36	6.46	0.062	0.32*	
	Free moment at peak breaking force		17.22	20.91	10.90	13.28	0.018	0.30*	
	Knee stiffness		9.18	6.74	14.48	11.27	0.092	0.79*	
Webster et al. [36]	vGRF	ACL	− 9.1	10.4	− 0.6	16.4	0.02	0.82*	
		Control group	1.9	15.2	1.7	14.4	N/A	0.01*	
	Knee joint moment	ACL	− 12.0	14.7	5.7	23.4	> 0.025	1.20*	
		Control group	3.8	25.5	3.5	21.9	N/A	0.01*	
	Hip joint moment	ACL	− 11.7	17.2	4.0	26.1	0.004	0.91*	
		Control group	− 6.5	20.2	2.8	26.6	N/A	0.46*	

*ACLR* anterior cruciate ligament rupture, *CON* concentric, *ECC* eccentric, *ES* effect size, *Hecc:Qcon* functional ratio of the peak eccentric torque of the hamstrings to the peak concentric torque of the quadriceps, *Kvert* vertical stiffness, *N/A* not available, *RSI* reactive strength index, *SD* standard deviation, *SLCMJ* single-leg countermovement jump, *SLDJ* single-leg drop jump, *vGRF* vertical ground reaction force

*value was calculated post-hoc by the authors

Asymmetries of GRF or related parameters were measured in eight studies. In the study of Bell et al. [7], no considerable effects of the loading protocol on vertical ground reaction force (vGRF) were found in males and females. Moreover, there was no meaningful interaction between time and sex. For loading rate asymmetry, a small effect was found for females but not males and for the interaction between time and sex. Bromley et al. [16] found large effects of a soccer match on eccentric impulse and peak force measured with SLCMJ. For concentric impulse, peak landing force, and peak landing impulse, only small or medium effect sizes were found.

In the study of Radzak et al. [35], significant changes in loading rate and free moment at peak braking force were found after a speed blinded protocol on a treadmill, however, with only small effect sizes. Moreover, the speed blinded protocol had a small effect on maximum adduction free moment, maximum absolute free moment, vertical stiffness, and maximum knee varus velocity, a medium effect on knee stiffness, and a large effect on knee internal rotation excursion.

Hanley and Tucker [31] assessed different parameters during a 10,000-m run on a treadmill, such as impact force, maximum force, or impulse. For impact force, small effect sizes were found between 1500 and 7500 m and medium effect sizes between 1500 and 9000 m. For maximum force, no effects were found either comparing the values successively or with the first measured section at 1500 m. For impulse, a small effect was found between 1500 and 7500 m. An effect size for the changes over the different time points could not be calculated due to missing values.

The study of Hodges et al. [32] revealed a significant main effect of repetitions on absolute average vGRF asymmetry and instantaneous %Left-Right vGRF asymmetry during the execution of barbell back squats, but only when initially highly symmetric subjects were removed. In this study, subjects became more symmetric within a set of squats. However, this was only an acute effect over the repetitions of one set, and the subjects started asymmetric again in the next set. Absolute peak instantaneous asymmetry (based on each foot’s instantaneous maximum GRF) and average %left-right vGRF asymmetry (calculated as the left GRFv% minus the right GRFv%) were not affected significantly by repetitions. Importantly, effect sizes were not stated and could not be calculated post-hoc due to missing values.

In the study of Webster et al. [36], subjects with former ACL rupture showed a more symmetric weight-bearing during the execution of squats. A large effect of the squat protocol on vGRF for the ACL group and no considerable effect for the control group was found. For the ACL group, there was also a large effect on knee and hip joint moment, but again no considerable (knee) or only a small (hip) effect in the control group.

Regarding parameters other than GRF, Bishop et al. [30] investigated the influence of a repeated sprint protocol on jump height measured with SLCMJs. They found a large effect on jump height asymmetry, but only between the last two sets compared to baseline. Jump height was also measured by Bromley et al. [16], who found a large effect of a soccer match on jump height asymmetry. In another study, Bishop et al. [38] also measured different parameters during SLCMJ (jump height, peak force, concentric impulse) and SLDJ (jump height, reactive strength index) before and after soccer matches. Although asymmetries were calculated, the exact values (means, standard deviations, effect sizes) were not provided. It was only stated that no meaningful changes were found on a group level and that the individual responses were highly variable.

Delextrat et al. [37] analyzed peak torque of the quadriceps, the hamstrings, and the functional ratio of the peak eccentric torque of the hamstrings to the peak concentric torque of the quadriceps (*H*_ecc_:*Q*_con_). The asymmetry values and effect sizes (calculated post-hoc) revealed no considerable effect of the protocol on peak torque of the hamstrings and small effects on the peak torque of the quadriceps and on the *H*_ecc_:*Q*_con_. Moreover, Mattes and Wolff [33] found significant changes for leg stretcher force with a main effect of the competition section on leg stretcher force. When considering separately by sex, no considerable effects were found for males and females.

Besides GRF, Hanley and Tucker [31] also measured spatio-temporal data, such as step length, step frequency, contact time, and flight time during a 10,000-m run. A medium effect was found when comparing the asymmetry values successively and to the 1500 m section. Only the contact time during the 10,000-m run changed with a small effect between 5000 and 7500 m.

Jordan et al. [21] found no significant changes for functional asymmetry values [21]. Girard et al. [20] found no significant changes for performance and running kinetics, running kinematics, or spring-mass characteristics. Similarly, Tucker and Hanley [34] reported that no significant changes were found for any of the measured variables. In none of the former three studies were effect sizes stated, nor could these be calculated post-hoc due to missing values. Regarding the size of asymmetries, it is noticeable that they vary a lot between the studies reaching from 0.33% [31] to 32% [16]. The values varied between the measured parameters, the used indices, and the points of time when they were measured (pre, post, or during the loading protocol).

## Discussion

The systematic review aimed to summarize the findings concerning the influence of exercise-induced fatigue on inter-limb asymmetries. Regarding the results in total, no explicit conclusions can be drawn as to whether exercise-induced fatigue influences asymmetries and increases the injury risk of an athlete. There are many diverging results due to the variation between the single studies. This was also found in similar systematic reviews or papers concerning the influence of fatigue on biomechanical parameters [8, 9, 41]. Therefore, the present results must be considered in more detail to infer recommendations for future research. In the following parts, the differences and consequences of the different study designs, loading protocols, tasks/procedures, and subjects will be discussed. Moreover, the outcome of the different studies will be analyzed to assess the potential influence of exercise-induced fatigue on inter-limb asymmetries. We will conclude with preliminary implications and directions for future research.

### Study Characteristics

#### Study Design

Two different kinds of study designs have been used: comparisons of pre-post measurements using separate tasks to investigate asymmetries before and after a load, and comparisons of asymmetries measured at different points of time during a loading protocol. These designs provide different perspectives of asymmetries under loading conditions: (1) an investigation of the influence of exercise-induced fatigue on a (sport) specific task disconnected from the movements during the physical load and (2) the consideration of possible changes directly connected with the physical load and its specific movements.

By using separate tasks pre and post, the influence of exercise-induced fatigue on a particular aspect of the sporting activity or the injury mechanism can be investigated. Importantly, however, the occurrence of asymmetries seems to depend on the chosen task and, therefore, should be analyzed with this in mind [7, 16, 19]. Measuring during a loading protocol enables analysis of how asymmetries change within a person over time. This might be helpful to find the point when asymmetries are likely to change because changes might occur progressively [23] and earlier than expected [9]. Furthermore, the level of effort might also influence asymmetries by leading to different internal loads [7]. Therefore, it could also be helpful to investigate how asymmetries change with different efforts and over the progression of an increasing physical load beyond the point an athlete might be fatigued. Furthermore, considering specific points of time after the physical load is suggested relevant as well to estimate how long changes in asymmetry persist and what this could mean for the injury risk and the recovery of an athlete [16].

Overall, a combination of both types of study designs seems appropriate to obtain more insights into asymmetries and possible occurring changes due to exercise-induced fatigue. This might help to answer how asymmetries change and why such changes are occurring. Moreover, it could be helpful to include a control group to learn more about the “real” influence of exercise-induced fatigue on inter-limb asymmetry and to measure asymmetries continuously to reveal possible changes over time.

#### Loading Protocols

Regarding the types of physical load, in all studies, different loading protocols were used to fatigue the subjects. The loading protocols had different durations and different termination criteria. Therefore, different external physical loads might lead to different internal loads, such as exercise-induced fatigue [1]. Also, the resulting internal loads depend on the individual athlete’s fitness level. Hence, the same external physical load might lead to different internal loads and a different amount of exercise-induced fatigue in different athletes [1]. This makes it difficult to compare the various loading protocols and studies, as suggested previously [8, 9, 41].

Moreover, it remains unclear if and how the different loading protocols stressed the subjects, and if a loading protocol has induced the same or a comparable amount of exercise-induced fatigue. The studies reviewed here used different methods to evoke and assess fatigue. This leads to the problem of different definitions, measurements, and operationalization of fatigue (see also [41]). A consistent approach is needed to improve research. According to Enoka and Duchateau [42], fatigue should be defined and regarded as the interaction of performance fatigability and perceived fatigability. Their approach includes physiological and psychological processes, but fatigue is only regarded as a general phenomenon and not divided into subcategories, e.g., central or peripheral fatigue. Benjaminse et al. [43] also describe fatigue as a complex interaction between psychological and physical factors. This interaction might influence the reactions to external stimuli and the decisions of an athlete in complex situations, e.g., landing or cutting in high-risk sports. The latter, in turn, might be associated with higher injury risk. However, the exact mechanisms behind the role of fatigue for injury risk are still unclear, and such a taxonomy needs greater evidence and application in research to become valid [41]. Moreover, athletes perceive fatigue differently, and other factors such as stress or recovery level may also influence fatigue [23].

#### Tasks/Procedures

Asymmetries were analyzed either with separate tasks/procedures or during the loading protocol as the given task. In both cases, the tasks were very different, and even within the same type of task, e.g., running, a considerable variability exists (durations, distances, or velocities). Besides, even when the same type of parameter, e.g., GRF, was measured, the results were also very different and inconsistent. This might be due to different internal loads and amounts of exercise-induced fatigue due to the different demands of the protocols. According to the different demands, different muscle groups are addressed, or the muscles are stressed differently. This again leads to different internal loads and different outcomes and results, even for the same parameter. Accordingly, task specificity of asymmetries should be taken into account during the selection of the tasks and protocols [7]. We recommend using tasks/procedures that reflect the sporting demands, e.g., SLCMJ, because unilateral landings depict one of the common injury mechanisms of non-contact injuries like ACL ruptures [8, 30]. Moreover, it should be considered that some tasks are not useful to detect large inter-limb asymmetries. For example, King et al. [44] showed that hop tests for distance seem to over-estimate an athlete’s rehabilitation status, especially when compared to vertical jump tests, which seem to be a better choice.

#### Subject Characteristics

Different groups of subjects with different characteristics were investigated. In total, more males were examined, although females are said to be more vulnerable to injuries like ACL ruptures suggesting that sex is an essential factor in this context [45]. Females have a two to ten times higher risk for injuries like ACL ruptures [45–47], and asymmetries are suggested as one possible explanation [12].

Moreover, the subjects also had different sporting backgrounds. In the context of sports, asymmetries are a phenomenon appearing in different kinds of sports [30]. Several sports are asymmetric in nature, e.g., soccer or handball, and promote the development of asymmetries [17]. Therefore, asymmetries might be a consequence of the sporting activity, rendering necessary that the sporting history of an athlete is considered [14–16]. In this context, the fitness level [13, 23] of an athlete might be relevant and influence the fatigue resistance and perception of an athlete [23]. Athletes with lower resistance to fatigue, for example, might fatigue earlier during physical load and show less optimal landing kinetics and kinematics [23]. Moreover, the volume of exposure to a specific sport and the familiarity with the investigated task might also influence the outcome. Maloney [13] suggests that asymmetries are an adaptive consequence magnifying due to longer participation in a specific type of sport.

In all studies, the subjects had to be healthy and injury-free at the time of the investigation. Two studies examined athletes with a former ACL rupture [21, 36]. Athletes with a previous injury often reduce the physical load on the affected side and use compensation mechanisms on the other side to maintain the performance [36]. Thus, they are still asymmetric, even if they are considered “healthy” (i.e., free of injury at the time of testing). Therefore, also the injury history appears an essential factor when assessing inter-limb asymmetries [9, 15].

All mentioned factors lead to different starting positions/baseline status of the subjects. In this context, little is known about the influence of exercise-induced fatigue on athletes without asymmetries or only small asymmetries at baseline [32], but this might be an interesting issue and give more information about which athletes are at a higher injury risk. Depending on the baseline value, athletes might react differently to a physical load, and there might be differences in the changes of asymmetries for athletes with a higher or lower baseline asymmetry value. Finally, the anthropometry of an athlete also influences the baseline level, for example, leg length discrepancy leads to asymmetries [15, 20, 32]. All these factors need to be either controlled by setting stricter inclusion/exclusion criteria or factored in analyses to better understand the role of asymmetries for injury risk.

### Other Potential Factors

Apart from the above, the variability and inconsistency in the results prompt that more factors might influence asymmetries and their changes in fatigued conditions. Therefore, the reasons for inter-limb asymmetries have to be investigated and considered [31]. The factors discussed below determine the primary position of an athlete and the reaction to a physical load.

#### Laterality

Laterality is assumed to influence the injury risk and the performance of an athlete [17]. Due to the asymmetric nature of sports [19] and because athletes have a preferred side to perform sport-specific actions like kicking or throwing [17, 48], athletes might be predisposed to the development of asymmetries [19]. According to Maloney [13], this task-specific preference, especially in sports, could be described as skill dominance. Additionally, he suggests differentiating such skill dominance from force dominance for given tasks, because asymmetry values differ if skill or force dominance is used as a reference. Therefore, a clear determination which leg is the dominant leg according to the athletic demands is essential [13]. Many studies equal a subject’s kicking leg with his/her dominant leg [49–51]. However, often this is not necessarily equivalent to the stronger or more skillful leg [52]. Also, in sports with repetitive and alternating movements, e.g., running or cycling, bilateral asymmetries are apparent [17]. One reason might be the different roles of the legs during movements: one is dominant for stabilization and support, and one for mobilization (e.g., propulsion or braking) and manipulation (e.g., kicking), altogether possibly causing inter-limb asymmetries [20, 35, 53]. Moreover, there may also be differences in the fatigability of the limbs [23, 30, 37, 54–56] and the direction of asymmetries [57]. Therefore, a consideration of laterality is advisable.

#### Limbs

It is also essential to differentiate between the lower and the upper limbs and to investigate both. Most of the research on inter-limb asymmetry has focused on the lower limbs, and in the current review, none of the included studies considered the upper body. The influence of asymmetries on the performance of the upper limbs has been investigated, e.g., in swimming [58], but less so in the case of injury prevention. Only a few studies were found regarding asymmetries of the upper limbs in this context (e.g., Corben et al. [56]), but none of them met the inclusion criteria. Indeed, asymmetries are not so often discussed as an influential factor for injuries of the upper limbs. This might be due to the main use of one limb during sporting actions, e.g., throwing or hitting, wherefore asymmetries might be present but have less influence on injuries or performance. Moreover, due to the nature of the upper limbs, the performance of one side is not directly affected by the other side. This makes asymmetries possibly less relevant, especially in asymmetric sports, e.g., handball or tennis. Nevertheless, many injuries in the upper limbs occur due to or during sporting activities, and it remains open whether asymmetries might also play a role in their emergence [59]. Therefore, asymmetries of the upper limbs should also be regarded, in sports with symmetric/cyclic actions of the upper limbs (e.g., swimming) as well as in sports with asymmetric actions of the upper limbs (e.g., handball, tennis).

#### Neuromuscular Aspects

Another point often discussed is that inter-limb asymmetries are not only mechanically based but also neurologically based [60]. Neuromuscular control is an important factor in the context of asymmetries and might be influenced by occurring fatigue [16]. In this context, movement strategies, especially during landings, are considered [21, 30, 31] because landings are one common mechanism for non-contact-injuries [8]. The landing strategies seem to change over time as the muscles became fatigued. In this regard, a loss of muscular control may lead to increased landing forces and increased injury risk [61–63]. Therefore, it seems important to consider neuromuscular deficits and changes to avoid injuries [21]. However, changes in neuromuscular strategies and patterns according to fatigue or increasing workload not only occur during landings but also during walking or cycling [35, 64]. Additionally, an athlete’s expertise might also influence their neuromuscular strategies. More experienced athletes might be able to use different neuromuscular strategies or to change their strategies, e.g., from quadriceps-dominant to ankle-dominant strategies during landing, to compensate for a loss of neuromuscular control due to asymmetries and fatigue and to maintain the level of performance [7, 30].

#### Calculation of Asymmetries

The equation used to calculate asymmetry is also important and influences the amount of the asymmetry. In all of the studies, an asymmetry value was calculated, but the calculation methods varied. This makes it difficult to compare the given values because there are differences in the equations, their reference values, and their results [65]. According to Bishop et al. [25], in the case of unilateral testing, the percentage difference (i.e., 100/ (max value) × (min value) × (− 1) + 100) should be used to calculate asymmetries. For bilateral tests, they recommend to use the Bilateral Asymmetry Index ((Dominant – Non-dominant)/(Dominant + Non-dominant) × 100) from Kobayashi et al. [66]. To obtain comparable results, we suggest using one of these equations depending on the type of task employed (i.e., unilateral or bilateral). Moreover, it might be helpful to regard not only the magnitude of asymmetry but also their direction to obtain insights on the underlying mechanisms and the influence of fatigue on limb dominance and asymmetries [57].

In the context of the amount of asymmetry, we also suggest that the (arbitrary) threshold needs to be scrutinized according to the variation between the different parameters [22]. According to a high variability (within the groups) and missing research with test-retest designs, the use of a concrete value to determine who has a higher risk for an injury is insufficient. Therefore, the “real” difference in function, strength, physical capacity, etc. between the limbs should be regarded. At least a more individual approach might be the best method to assess the injury risk of an athlete [67] because group means do not provide sufficient information [57]. In this respect, intra-limb variability should be considered, and the coefficient of variance of the test should also be calculated to quantify if the measured asymmetry is “real” [25, 68]. Moreover, longitudinal data are needed to detect changes over time [25, 57] as it has been done by Bishop et al. [38].

Altogether, the given results are highly inconsistent, just as in related reviews of Barber-Westin and Noyes [8] or Santamaria and Webster [9]. This is attributed to task specificity of asymmetries, the different loading protocols, or the way that asymmetry was quantified. Therefore, we suggest that not only one parameter should be considered, but rather a combination of different parameters is necessary to consider an athlete as asymmetric [31] and to assess the injury risk of an athlete [16]. The tasks/procedures should be adapted to the given sporting demands of the sport an athlete is competing in (e.g., intensity, duration) and to its specific movements and requirements. Finally, limb dominance and fatigue should be clearly defined and assessed, and a clear concept for the calculation of asymmetries is necessary to improve research and render findings comparable.

## Conclusion

In view of the results and the formerly discussed points, a clear statement on the influence of exercise-induced fatigue on inter-limb asymmetries is difficult. The main reason for the inconsistent and diverging results is that no clear systematization exists in current research concerning asymmetries and physical load. The study characteristics are so heterogeneous that it is difficult to compare the studies and to derive explicit conclusions about the influence of exercise-induced fatigue on asymmetries and the relation to the injury risk of an athlete. Future research needs to implement a systematic research program, considering the above-discussed aspects.

Regarding the design of the studies, based on the systematic review, we suggest a combination of the two different types of designs (pre/post and during). First, pre-post designs should be used to assess asymmetries with sport-specific tasks. Then, as a second step, an observation over the progression or at different points of time during physical load should follow. Moreover, longitudinal studies are needed to provide more information on long-term changes of asymmetries.

Concerning the selection of protocols and tasks, it cannot be stated which type of loading protocol should be used. This depends on the context or aim of the study or assessment. Nevertheless, according to the nature of inter-limb asymmetries, it seems plausible to create a design that reflects the real sporting situation and its demands [23]. Additionally, inter-limb asymmetries could occur in many different movements or actions [30]. Therefore, to consider an athlete as asymmetric and to assign him or her the degree of injury risk, it is useful to examine different parameters.

**Fig. 2:**
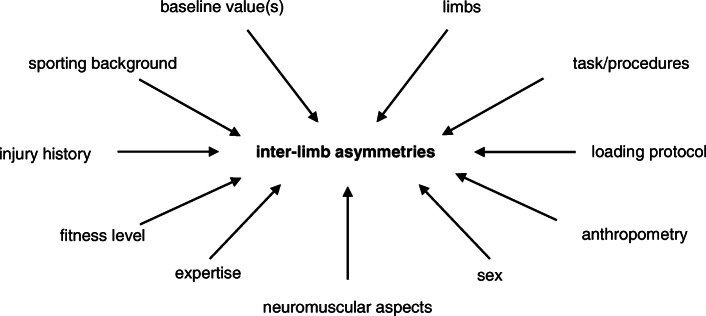
Factors that could influence inter-limb asymmetries

To systematize the research, we suggest the implementation of laboratory studies using, for example, running protocols and the use of cutting or landing movements to depict “real” sporting demands. Moreover, we recommend unilateral (when possible) and bilateral testing, to avoid as well as to assess that one side is compensating for the other and to reflect the common injury mechanisms. Moreover, the construction of study design, the selection of subjects, and the analysis should consider different factors such as sex, sporting background, injury history, or anthropometric factors like leg length, which all might influence asymmetry. In addition, the analyses should concern an individual athlete’s reaction to physical load (i.e., the internal load) and the occurrence of exercise-induced fatigue (Fig. 2). Future research could also benefit from a more individual approach and the inclusion of a control group to obtain more insights into the “real” influence of fatigue.

Finally, the above-mentioned steps could also be implemented in research programs targeting asymmetries of the upper limbs, which have almost been neglected in the context of sports injuries.

## Data Availability

All data supporting the findings of this review are available in this article.

## Abbreviations

ACL: Anterior cruciate ligament
CON: Concentric
ECC: Eccentric
GRF: Ground reaction force
PRISMA: Preferred Reporting Items for Systematic Reviews
SLCMJ: Single-leg countermovement jump
SLDJ: Single-leg drop jump
RPE: Rating of perceived exertion
RSA: Repeated sprint ability
vGRF: Vertical ground reaction force
